# Supplementary material for: Antimicrobial, Anti-Biofilm, Anti-Quorum Sensing and Cytotoxic Activities of Thymbra spicata L. subsp. spicata Essential Oils
Source: Antibiotics (Basel). 2025 Feb 11;14(2):181. doi: 10.3390/antibiotics14020181 (PMC11851892; doi:10.3390/antibiotics14020181)
Supplement: Supplementary file 1 [file antibiotics-14-00181-s001.zip › antibiotics-3437544-supplementary.pdf]

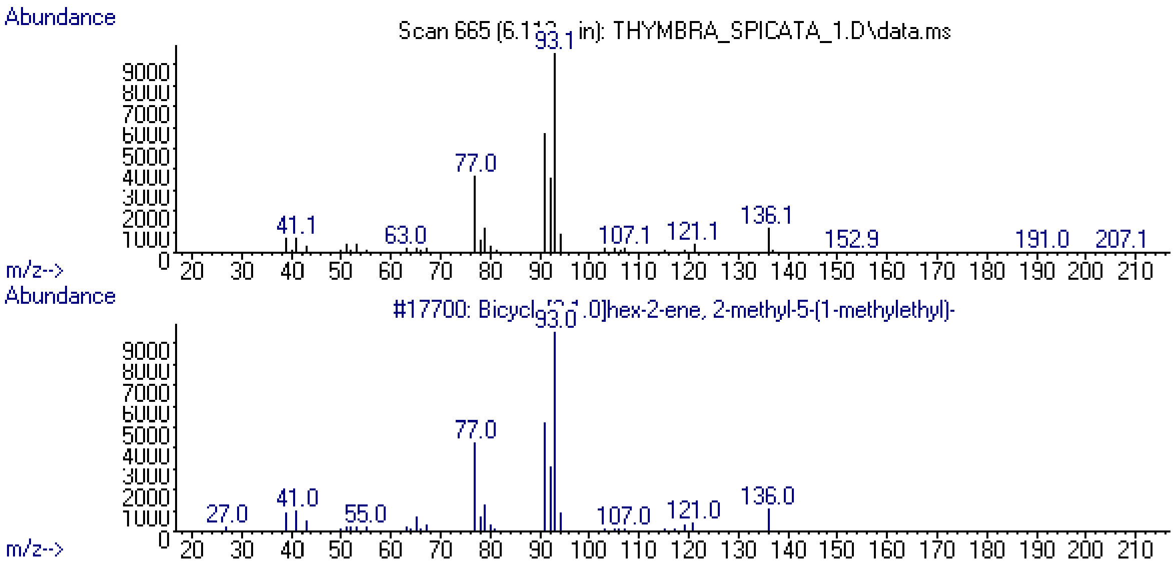

Figure S1. Mass spectrum of α-Thujene

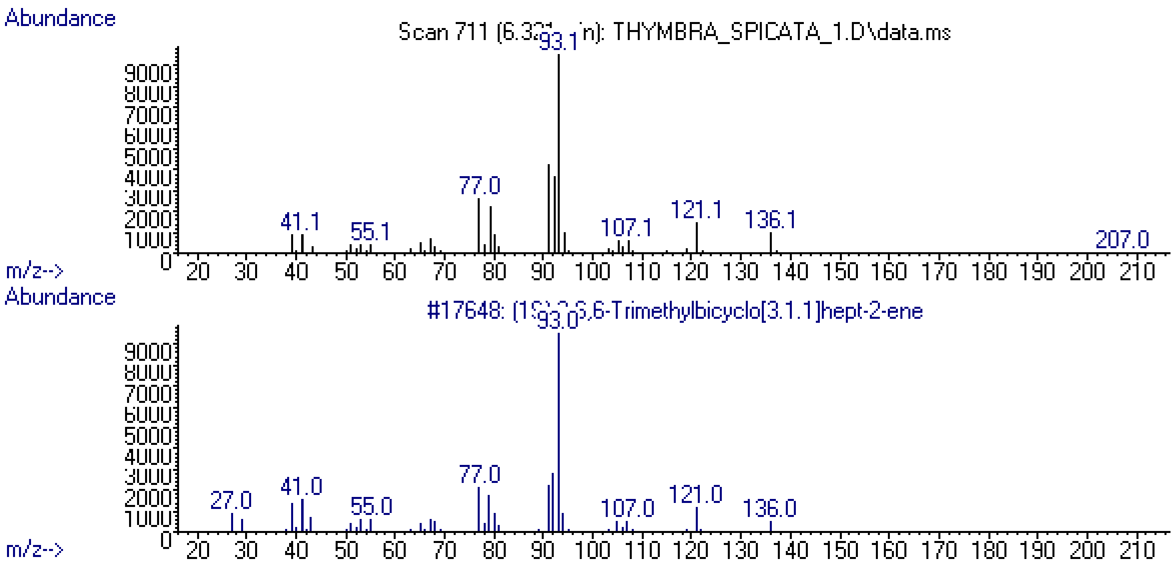

Figure S2. Mass spectrum of α-Pinene

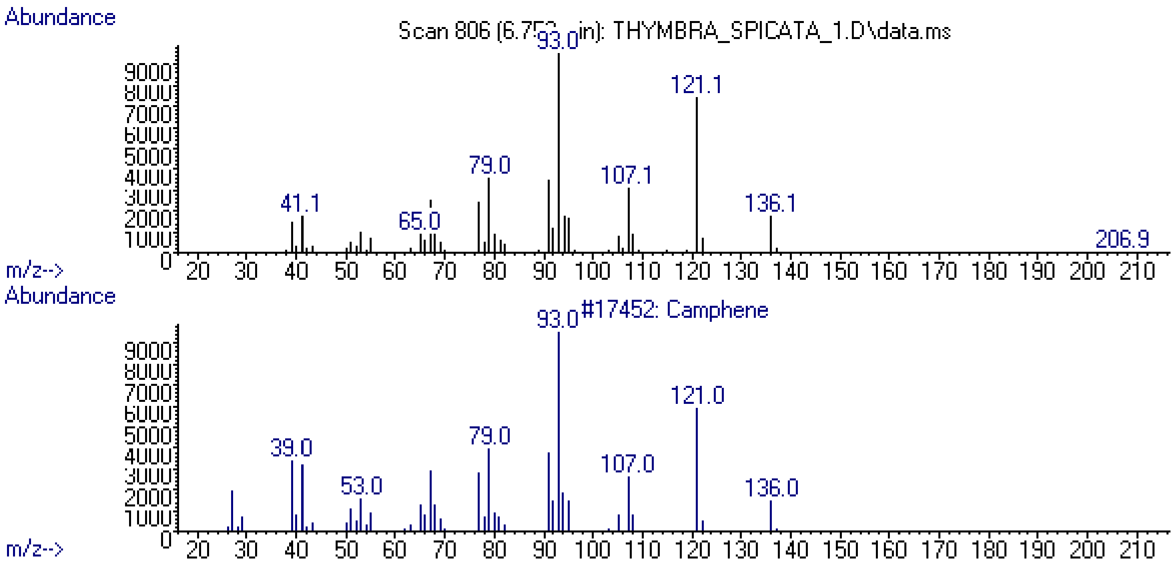

Figure S3. Mass spectrum of Camphene

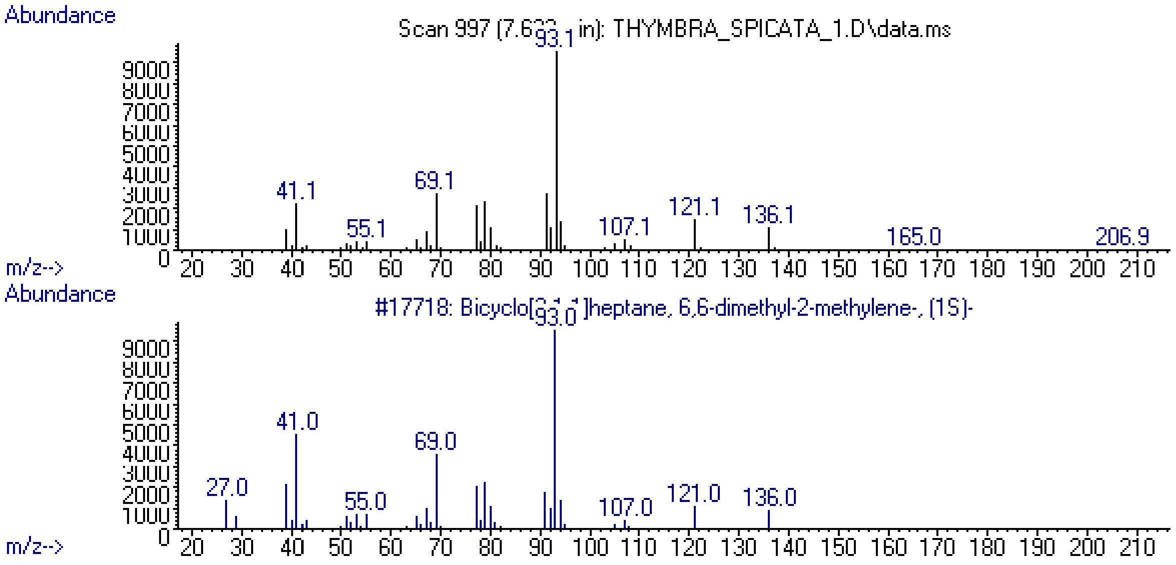

Figure S4. Mass spectrum of β-pinene

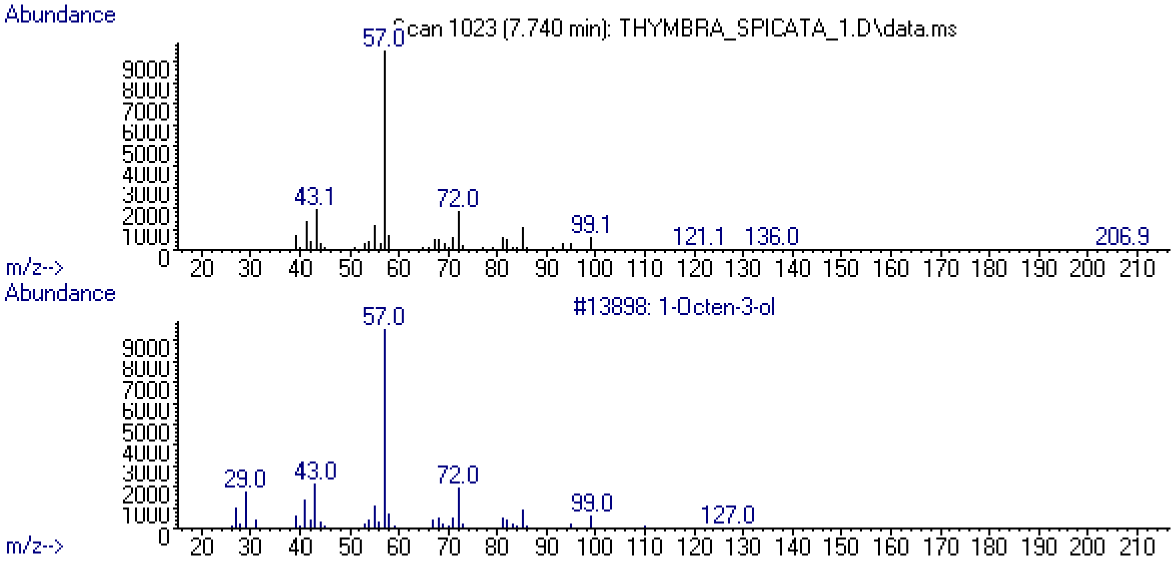

Figure S5. Mass spectrum of 1-Octen-3-ol

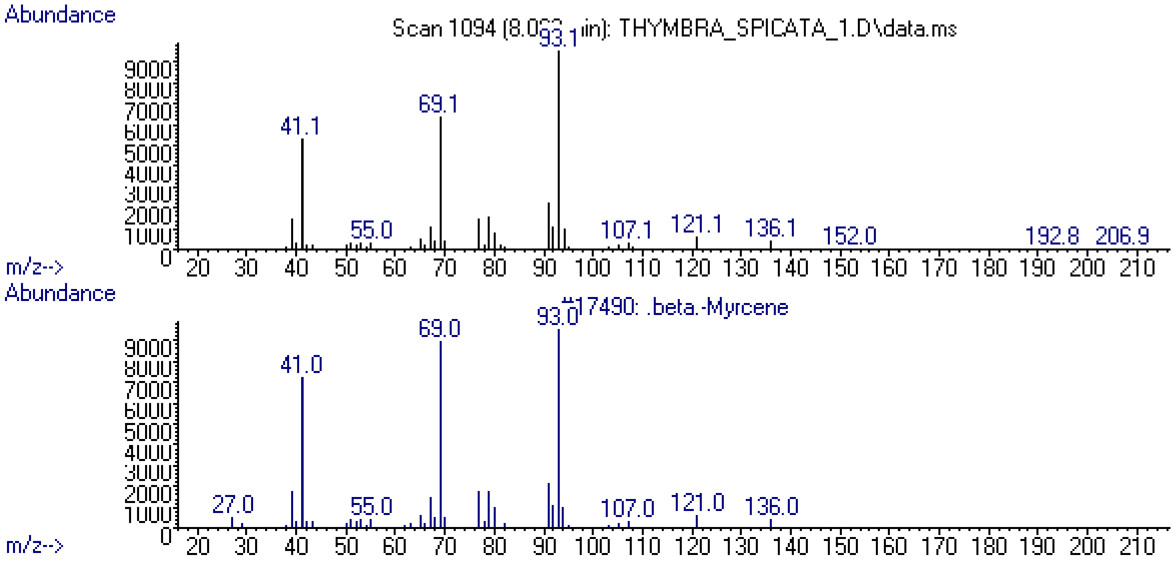

Figure S6. Mass spectrum of β-Myrcene

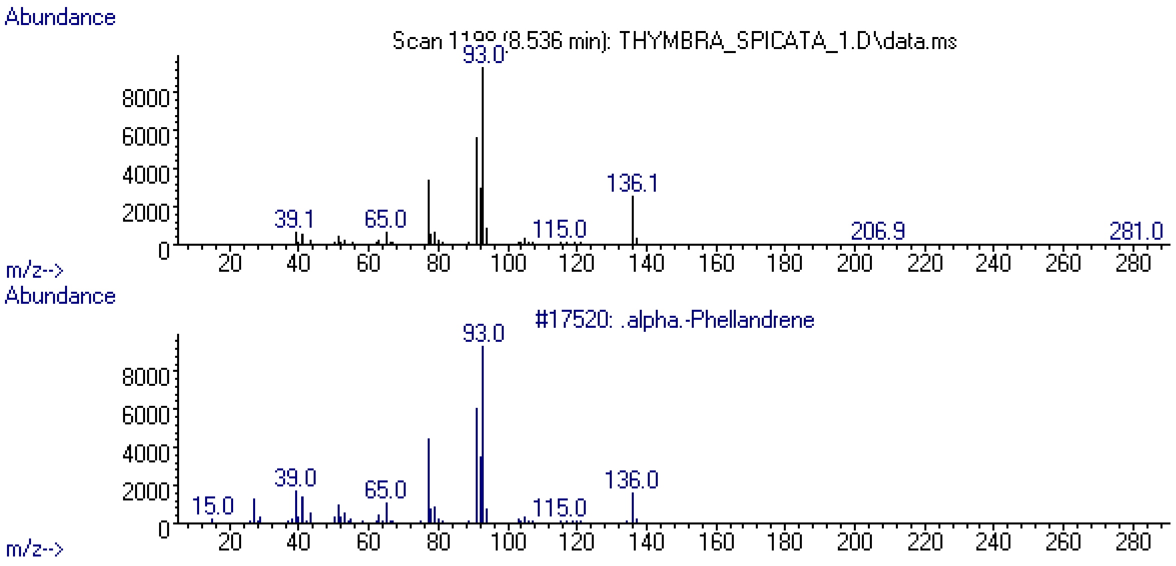

Figure S7. Mass spectrum of α-Phellandrene

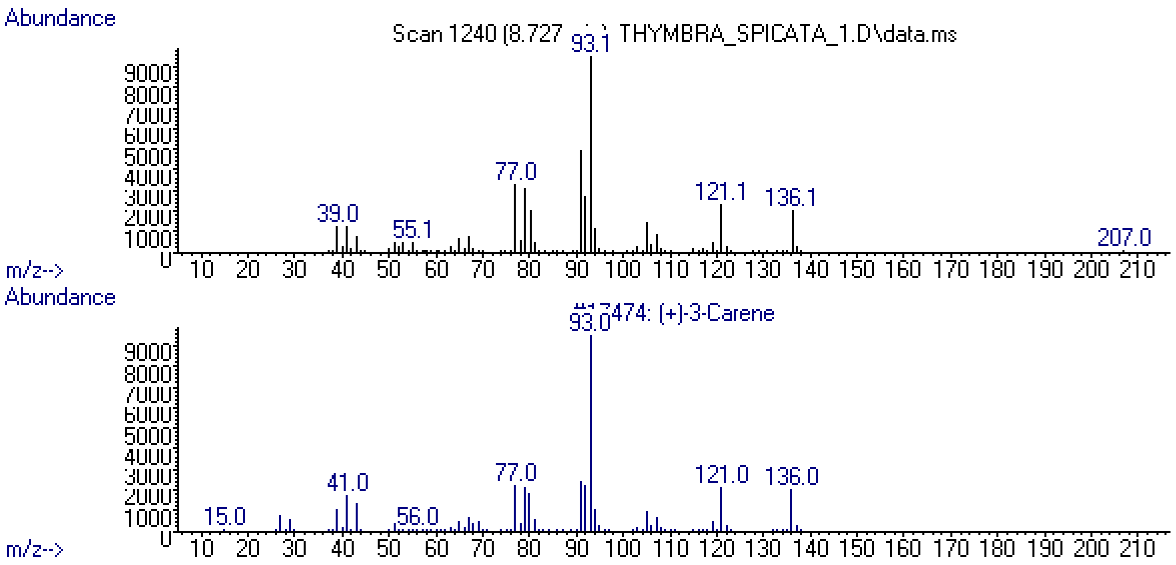

Figure S8. Mass spectrum of δ-3-carene

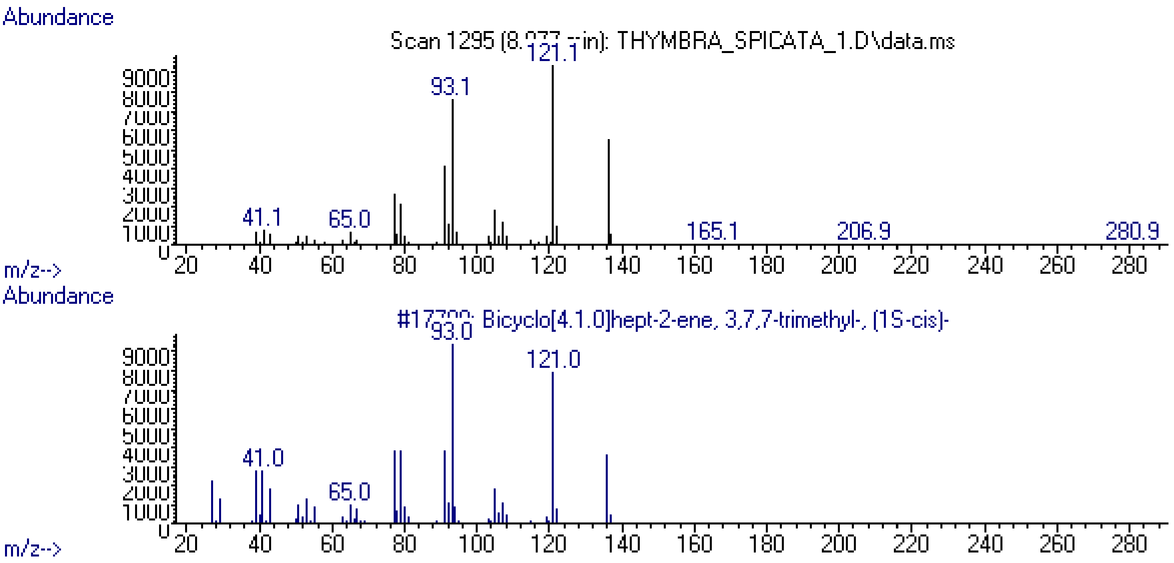

Figure S9. Mass spectrum of α-Terpinene

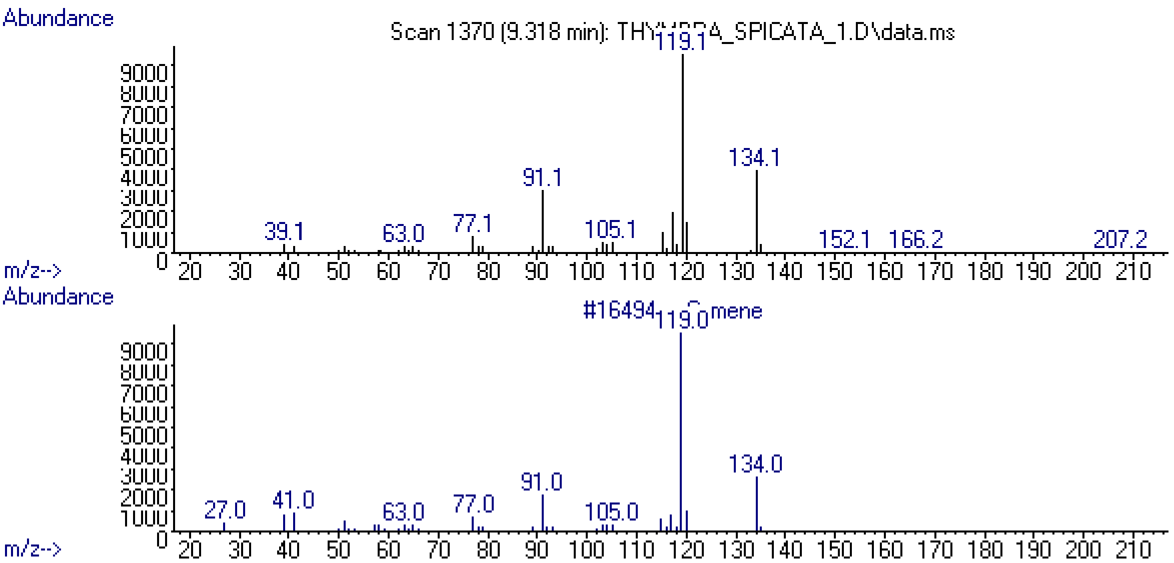

Figure S10. Mass spectrum of p-Cymene

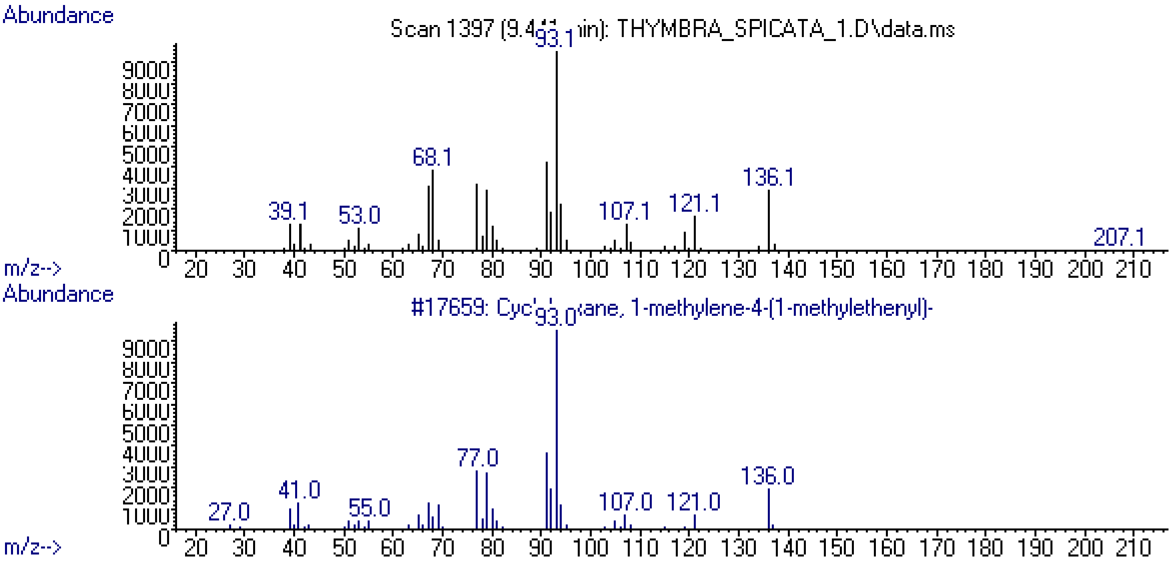

Figure S11. Mass spectrum of β-Phellandrene

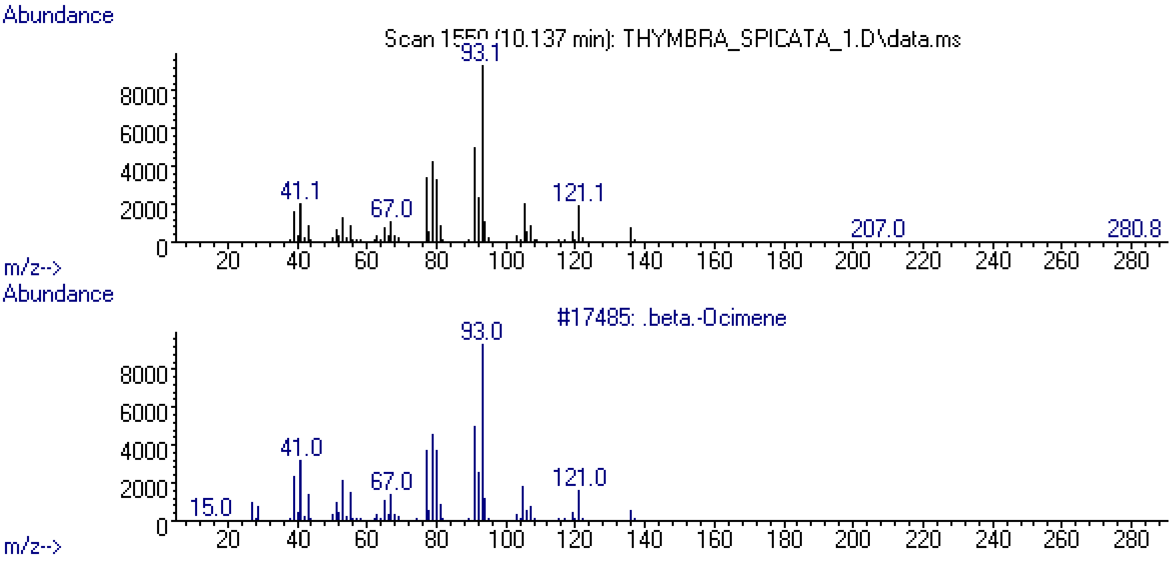

Figure S12. Mass spectrum of β-cis-Ocimene

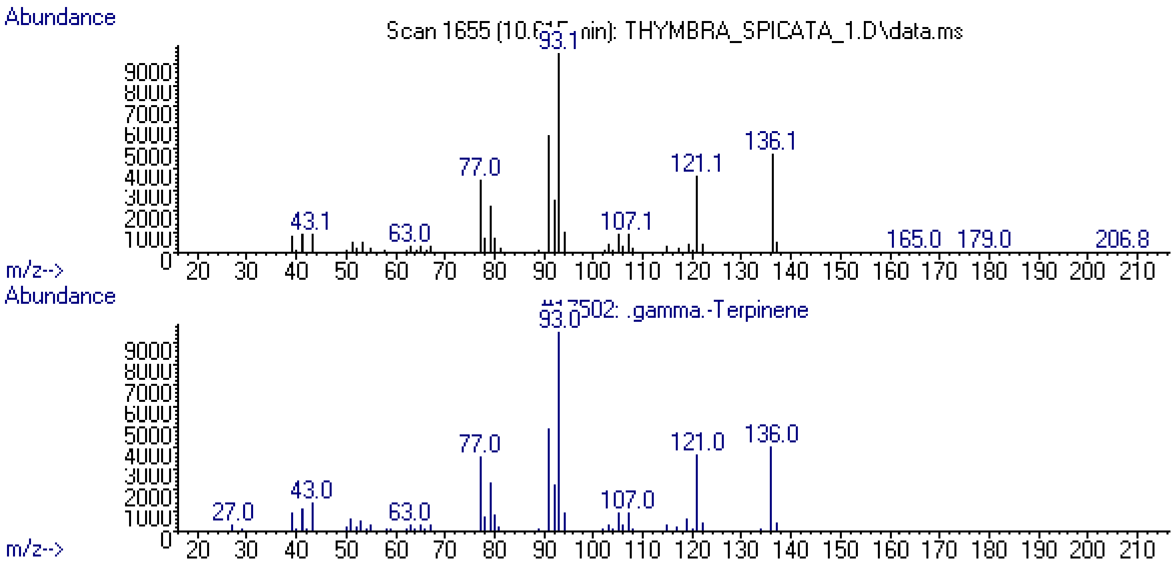

Figure S13. Mass spectrum of γ-Terpinene

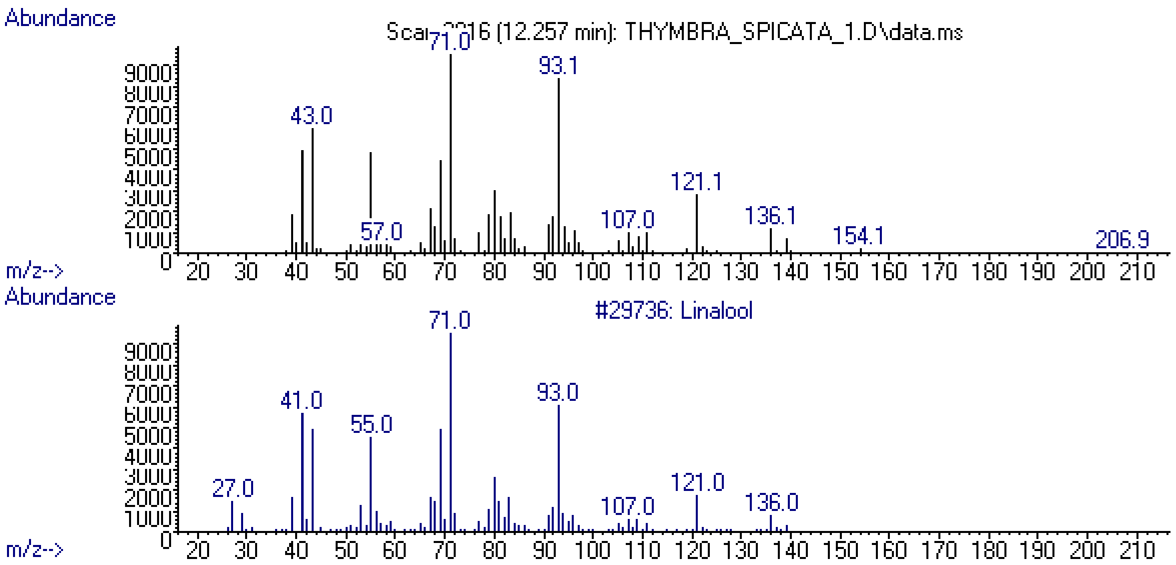

Figure S14. Mass spectrum of Linalool

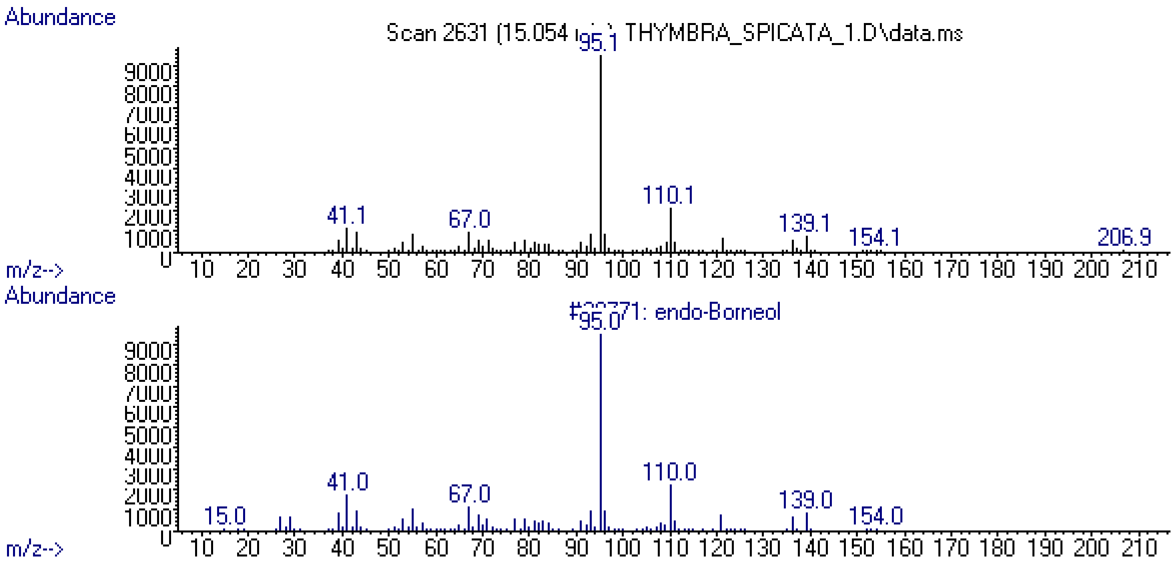

Figure S15. Mass spectrum of endo-Borneol

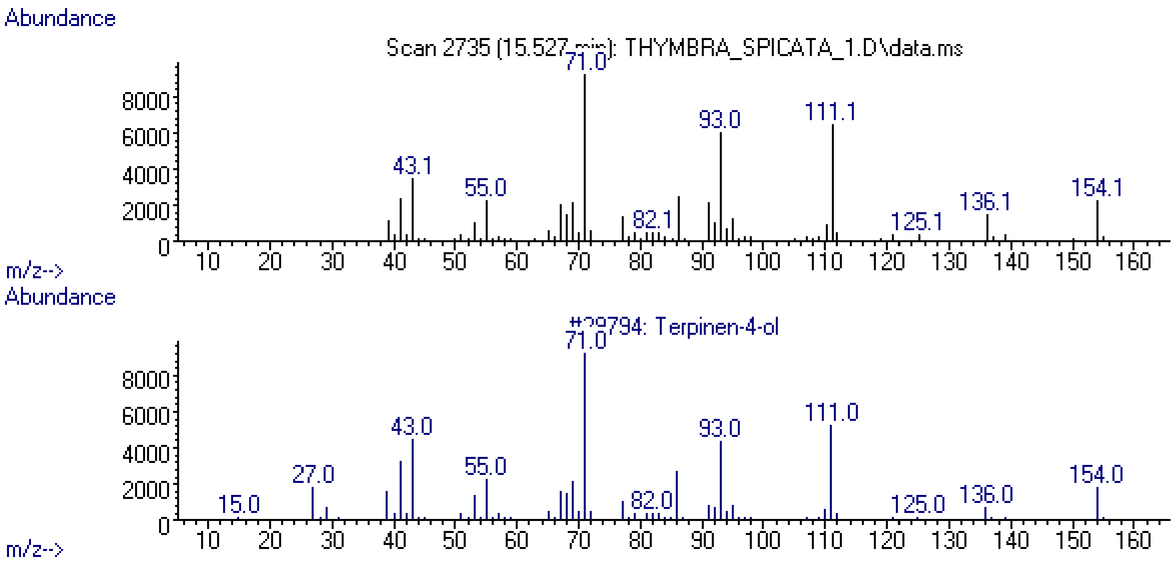

Figure S16. Mass spectrum of Terpinen-4-ol

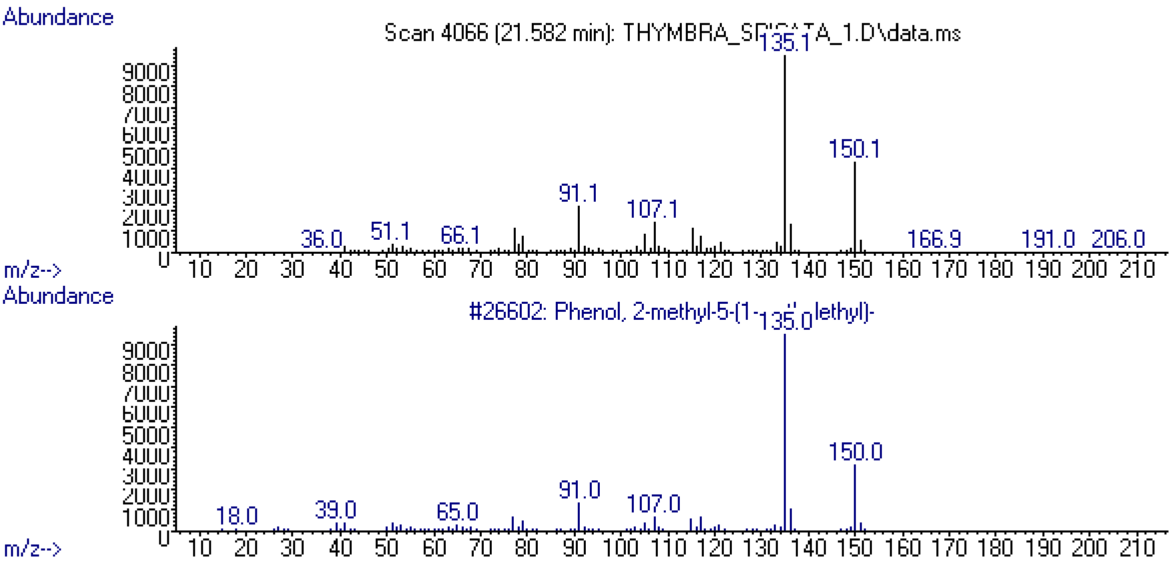

Figure S17. Mass spectrum of Carvacrol

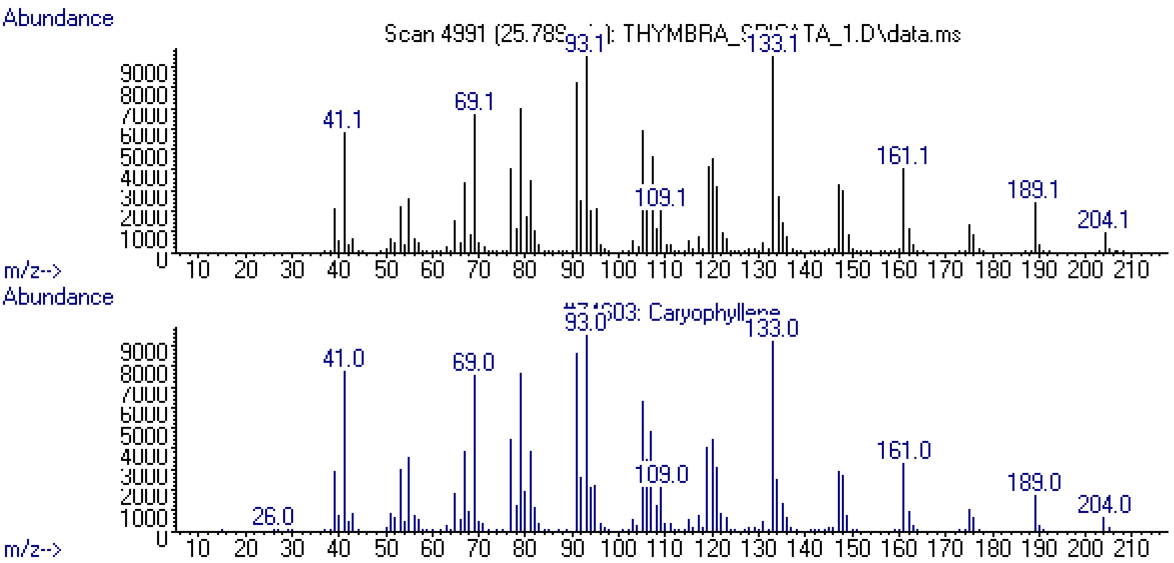

Figure S18. Mass spectrum of Caryophyllene

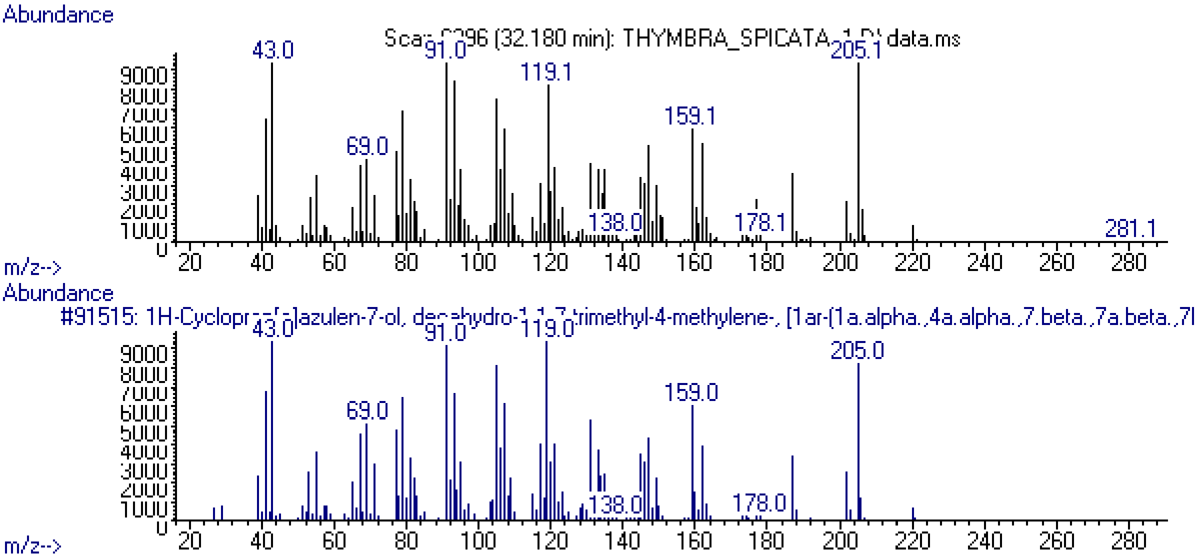

Figure S19. Mass spectrum of Spathulenol

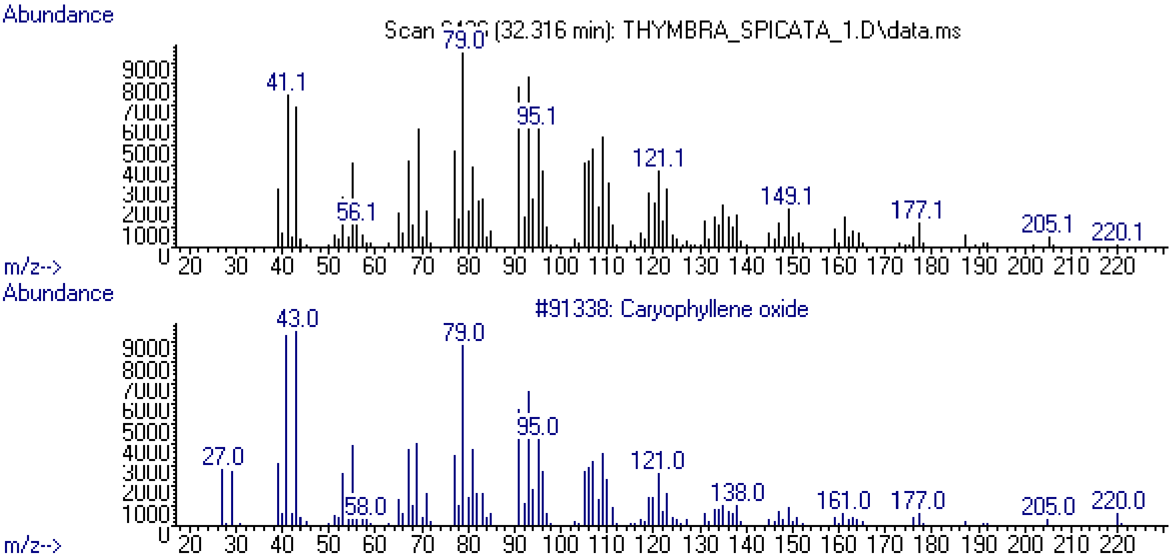

Figure S20. Mass spectrum of Caryophyllene oxide

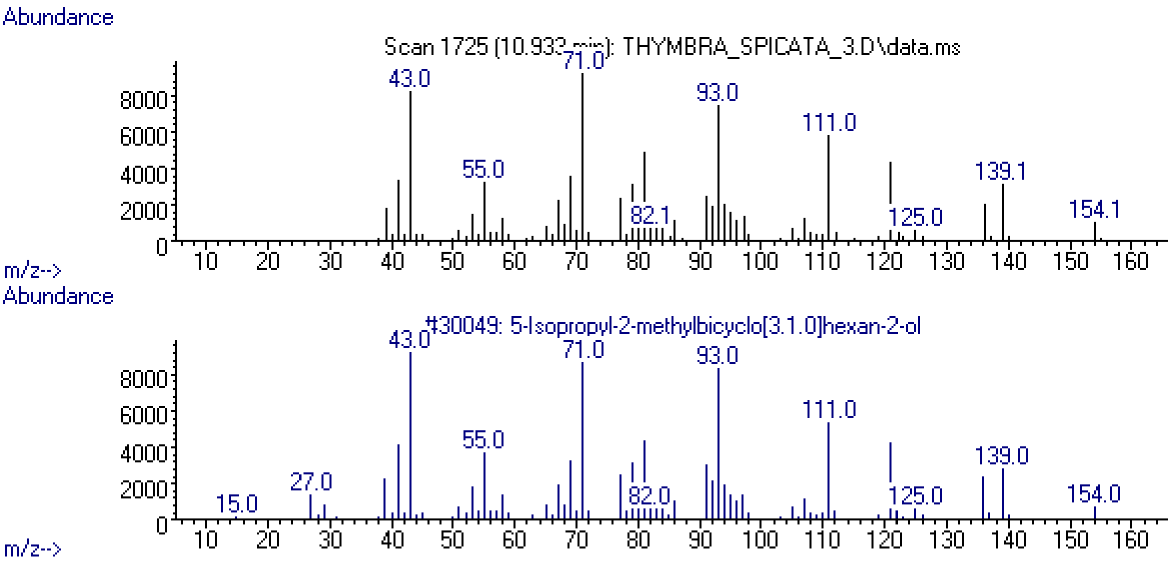

Figure S21. Mass spectrum of Trans-Sabinene hydrate

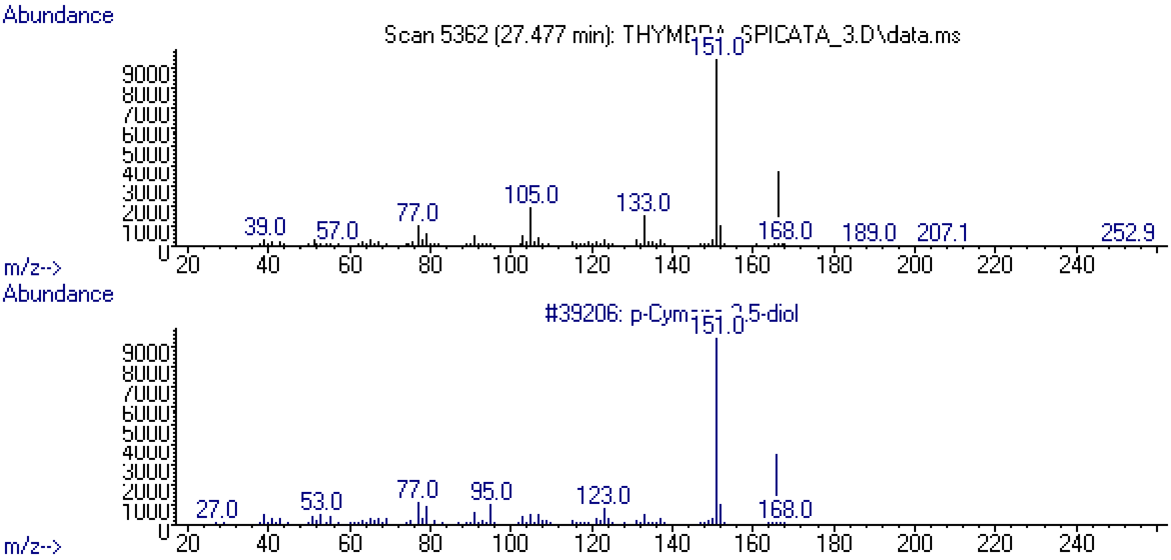

Figure S22. Mass spectrum of p-tert-Butylcatechol

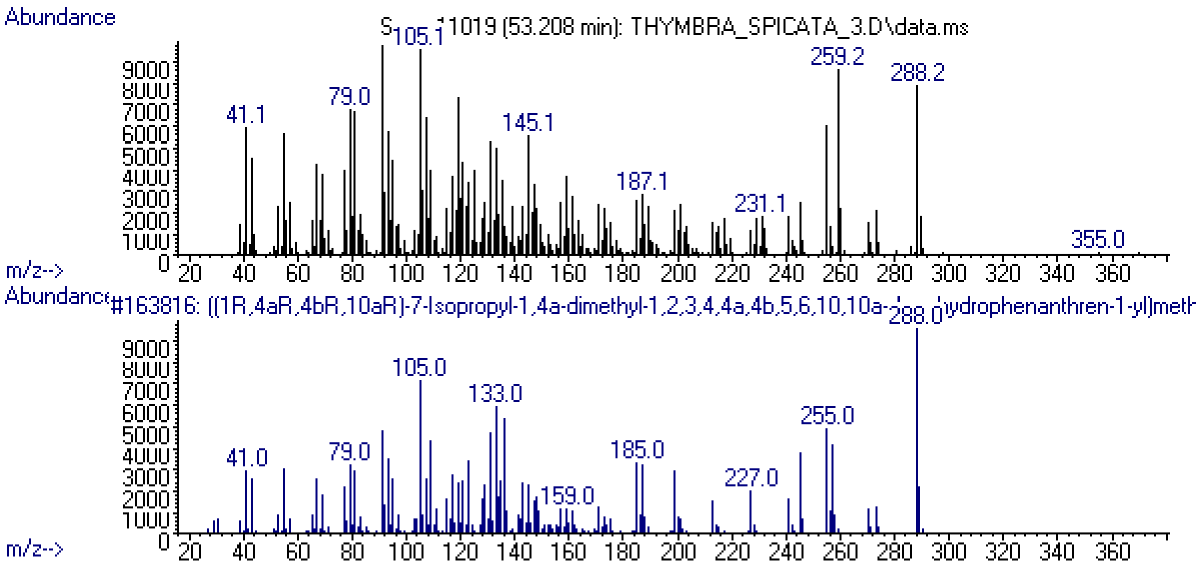

Figure S23. Mass spectrum of Pimara-7,15-dien-3-ol
